# Neuroinflammation and Depression: Microglia Activation, Extracellular Microvesicles and microRNA Dysregulation

**DOI:** 10.3389/fncel.2015.00476

**Published:** 2015-12-17

**Authors:** Dora Brites, Adelaide Fernandes

**Affiliations:** ^1^Research Institute for Medicines (iMed.ULisboa), Faculty of Pharmacy, Universidade de LisboaLisbon, Portugal; ^2^Department of Biochemistry and Human Biology, Faculty of Pharmacy, Universidade de LisboaLisbon, Portugal

**Keywords:** astrocytes, exosomes, ectosomes, microglia, neurodegeneration, glia interplay, oligodendrocytes, microRNAs

## Abstract

Patients with chronic inflammation are often associated with the emergence of depression symptoms, while diagnosed depressed patients show increased levels of circulating cytokines. Further studies revealed the activation of the brain immune cell microglia in depressed patients with a greater magnitude in individuals that committed suicide, indicating a crucial role for neuroinflammation in depression brain pathogenesis. Rapid advances in the understanding of microglial and astrocytic neurobiology were obtained in the past 15–20 years. Indeed, recent data reveal that microglia play an important role in managing neuronal cell death, neurogenesis, and synaptic interactions, besides their involvement in immune-response generating cytokines. The communication between microglia and neurons is essential to synchronize these diverse functions with brain activity. Evidence is accumulating that secreted extracellular vesicles (EVs), comprising ectosomes and exosomes with a size ranging from 0.1–1 μm, are key players in intercellular signaling. These EVs may carry specific proteins, mRNAs and microRNAs (miRNAs). Transfer of exosomes to neurons was shown to be mediated by oligodendrocytes, microglia and astrocytes that may either be supportive to neurons, or instead disseminate the disease. Interestingly, several recent reports have identified changes in miRNAs in depressed patients, which target not only crucial pathways associated with synaptic plasticity, learning and memory but also the production of neurotrophic factors and immune cell modulation. In this article, we discuss the role of neuroinflammation in the emergence of depression, namely dynamic alterations in the status of microglia response to stimulation, and how their activation phenotypes may have an etiological role in neurodegeneneration, in particular in depressive-like behavior. We will overview the involvement of miRNAs, exosomes, ectosomes and microglia in regulating critical pathways associated with depression and how they may contribute to other brain disorders including amyotrophic lateral sclerosis (ALS), Alzheimer’s disease (AD) and Parkinson’s disease (PD), which share several neuroinflammatory-associated processes. Specific reference will be made to EVs as potential biomarkers and disease monitoring approaches, focusing on their potentialities as drug delivery vehicles, and on putative therapeutic strategies using autologous exosome-based delivery systems to treat neurodegenerative and psychiatric disorders.

## Introduction

Depression (major depressive disorder, MDD) is a mood disorder of multifactorial origin, including genetic and environmental factors, estimated to affect 350 million people worldwide (Jo et al., 2015). It is responsible for increased morbidity and mortality, adverse health behaviors, lost work productivity and increased health care utilization (Benton et al., 2007). Suicide accounts for almost 1 million lives lost each year, i.e., 3000 suicide deaths per day (World Health and Organization, 2012). About 25% of people diagnosed with MDD are under 19 years old and about 40% of patients do not adequately respond to current therapy (Dwivedi, 2014). To that it may account the poor understanding of the underlying mechanisms leading to MDD and suicidal behavior.

Stress, impaired neurogenesis and defects in synaptic plasticity represent three interconnected factors that are associated with depression (Dwivedi, 2011; Mouillet-Richard et al., 2012). Characteristic pathophysiological hallmark features include monoamine depletion, down-regulation of neurotrophin signaling, and glucocorticoid receptor (GR) resistance, as well as excess of glutamate, corticotrophin-releasing hormone and cortisol levels (Carvalho et al., 2015; Jo et al., 2015). Stress has been associated to the development of clinical depression, and evidence from preclinical studies suggests a role of microglia in depression and stress. Most curious, chronic stress has been known to promote microglial hyper-ramification and astroglial atrophy (Tynan et al., 2013), as well as lower immunoreactivity of myelin basic protein (MBP) and fewer mature oligodendrocytes (Yang et al., 2015) in the prefrontal cortex of rodents. Other molecules that are gaining attention in depressive pathophysiology are the cyclic adenosine monophosphate (cAMP) response element binding protein (CREB) involved in learning and memory, whose expression is decreased in depressed patients, and the vascular endothelial growth factor (VEGF), an angiogenic cytokine, with decreased mRNA levels in peripheral leukocytes of such individuals (Dwivedi, 2009).

As mentioned above, stress is a major risk factor for depression leading to the activation of hypothalamus-pituitary-adrenal (HPA) axis, as well as to a reduced hippocampal neurogenesis, together with impaired hippocampal synaptic plasticity and morphological neuroplasticity. Post-mortem studies revealed unique pathological alterations in neuronal and glial cells in the dorsolateral prefrontal cortex of patients with MDD, in particular a reduced cell size and density (Stockmeier and Rajkowska, 2004). Astrocyte deficits such as a decreased cell number of glial fibrillary acidic protein (GFAP)—immunoreactive cells and oligodendrocyte pathology, probably related to disruptions of white matter tracts, were found in depressed individuals (Rajkowska and Miguel-Hidalgo, 2007). Abnormal activation of microglia, the immunologic guardian cells of the brain, and increased microglial cell numbers were observed in depression and in anxiety disorders, although it is yet unclear how it relates with psychopathological conditions (Serafini et al., 2015). To note that reactive glial cells are sources and targets of various inflammatory cytokines, and as so are involved in the regulation of neuroinflammation and tissue repair (Rajkowska and Miguel-Hidalgo, 2007). Lately, depression has been described as a microglia-associated disorder, and besides the excessive cell activation and increased cell number, microglia decline and senescence was observed in some depressed patients as well (Yirmiya et al., 2015).

Recent evidences suggest that extracellular vesicles (EVs) secreted by neurons and glia, including endosome-derived exosomes and fragments of the cellular plasma membrane play a key role in intercellular communication and neuroinflammation by transporting messenger RNA (mRNA), microRNA (miRNA) and proteins (Pegtel et al., 2014). EVs are recognized as having a role in pathogenesis and dissemination of inflammatory diseases, besides their potential as biomarkers and therapeutic vehicles (Buzas et al., 2014). Reactive microglia were shown to release exosomes and microvesicles (MVs) carrying the pro-inflammatory cytokine interleukin-1β (IL-1β), the IL-1β-processing enzyme caspase-1, and the P2X7 receptor that may induce and propagate inflammatory reactions throughout the brain (Frühbeis et al., 2013).

A number of treatments were developed to increase the availability of monoamines, such as serotonin, due to a shift from serotonin to kynurenine pathway in tryptophan catabolism. In the central nervous system (CNS) the kynurenine pathway is mediated by astrocytes, microglia and infiltrating macrophages (Jo et al., 2015). Current therapies usually result in relapse rates of only 50%, reason why a better understanding of the pathomechanisms involved in MDD may help in the discovery of more effective and cost-effective treatment alternatives. Accumulating evidence suggests that glial pathology and the decrease in the number of glial cells are prominent features in MDD (Hamidi et al., 2004; Altshuler et al., 2010). Glial reduction in tissue samples from subjects diagnosed with MDD, at least in amygdala, was shown to be due to a loss of oligodendrocytes once no significant changes were observed on microglia or astrocytes (Hamidi et al., 2004). Patients with depression have been shown to evidence increased serum levels of pro-inflammatory cytokines that returned to normal by treatment with antidepressants for 3 months (Dahl et al., 2014). Some studies revealed that the reinforcement of neurotrophin expression and stimulation of neurogenesis by causing antidepressant-like effects may be of therapeutic relevance in chronically depressed patients (Van Buel et al., 2015). These Authors argue that targeted potentiation, instead of suppression of neuroinflammation, may be of therapeutic relevance in chronic depressed patients. Interestingly, electroconvulsive therapy of MDD was indicated to facilitate the action of antidepressants by inducing hippocampal neurogenesis through the modulation of microglial activation (Rotheneichner et al., 2014). In addition, minocycline, which is a suppressor of activated microglia, has been shown to exert protective effects by reducing microglial activation, oxidative stress and inflammation (Réus et al., 2015). Therefore, there are some controversial hypotheses on the best therapeutic approaches to MDD.

This review article aims to summarize data about the effects of immune system dysregulation and microglial activation on mood dysregulation and will also discuss the role of EVs and their specific cargo, namely miRNAs, as means by which these neuroinflammatory mechanisms take place and influence neighboring cells leading to the propagation of inflammation.

## Inflammation-Associated Depression

Chronic inflammation in physically ill patients is often associated with the development of symptoms of depression (Benton et al., 2007; Goldberg, 2010). Activation of the peripheral immune system leads to increased cytokine levels that are actively transported into the CNS stimulating astrocytes and microglial cells, which in turn produce cytokines by a feedback mechanism (Müller and Ackenheil, 1998). In such condition there is sickness exacerbation and the development of symptoms of depression in susceptible patients (Dantzer et al., 2008). Although not completely clarified the intracellular molecular mechanisms linking inflammation and depression, it was demonstrated that microglia besides releasing inflammatory mediators also secrete glutamate and metabolize kynurenine transported to the CNS into quinolinic acid, a neurotoxic compound (Dantzer and Walker, 2014). Astrocytes seem not to be able to uptake the excess of glutamate that together with quinolinic acid will enhance glutamatergic neurotransmission leading to the development of symptoms of depression. Proinflammatory cytokines can additionally stimulate HPA axis to release glucocorticoids that supress neurogenesis (Liu et al., 2003). Recently, it was suggested that neuroinflammation may not only derive from pathological conditions, but also from enhanced neuronal activity denominated as neurogenic neuroinflammation that may aggravate stressful stimuli (Xanthos and Sandkuhler, 2014). How inflammation decreases neurogenesis and leads to dysfunction of neurotrophic system is scarcely understood as it is the cross-talk between microglia, mostly associated to neuroinflammation, and astrocytes that produce neurotrophins (Song and Wang, 2011).

It was observed that chronic unpredictable mild stress-exposed rats, a well-documented model of depression, produced increased IL-1β mRNA and protein levels in the prefrontal cortex, which were not reproduced in serum or cerebrospinal fluid (CSF). Nuclear factor kappa B (NF-κB) inflammatory pathway and nucleotide binding and oligomerization domain-like receptor family pyrin domain-containing 3 (NLRP3)-inflammasome activation in microglial cells revealed to be implicated (Pan et al., 2014). However, there are controversial data on IL-1β alterations in periphery and CSF between depressed animals and patients. In a meta-analysis study, no changes were found for IL-1β, though elevated levels of tumour necrosis factor (TNF)-α_and IL-6 were observed in depressed subjects compared with control subjects (Dowlati et al., 2010). In other studies, patients with depression showed increased serum levels of IL-1β, IL-6, IL-8, IL-12 and TNF-α, together with a decrease in IL-10 levels, an anti-inflammatory cytokine (Schiepers et al., 2005; O’Brien et al., 2007; Song et al., 2009). Actually, studies point to the activation of inflammatory responses and to microglial P2X7, a purinergic ion channel activated by ATP, as contributors to the pathogenesis of depression (for review, see Stokes et al., 2015). Microglia activation is particularly enhanced in individuals who committed suicide and in depressive patients (Steiner et al., 2008; Schnieder et al., 2014). In addition, suicide has been related not only to microglial activation, but also to an increase of perivascular macrophages around blood vessels (Torres-Platas et al., 2014). In fact, stress has been linked to the development of both depression and anxiety, with a key contribution of microglia activation, as well as of recruitment of peripheral macrophages into the brain to such events (Phillips et al., 2015; Réus et al., 2015). On the other hand, the neuroinflammatory status associated with depression-like symptoms may also result from the existence of peripheral or central chronic inflammatory processes that continuously activate peripheral macrophages, sending inflammatory signals to the brain (Roman et al., 2013).

Hippocampus is a region with a high density of microglial cells, especially in the CA1 region, and hippocampal microglial activation demonstrated to be originated by stress and suggested to be implicated in the pathophysiology of MDD, as well as in other psychiatric and stress-related disorders (Walker et al., 2013). Increased high mobility group box-1 (HMGB-1) protein was demonstrated to be increased in the hippocampus of male Sprague Dawley rats after tail shocks and to be responsible for microglia priming by acting on the NLRP3 inflammasome (Weber et al., 2015). In addition, low dose administration of lipopolysaccharide (LPS) to mice, as a model of depression, led to HMGB1 translocation from the nucleus to the cytoplasm, while blockage of HMGB1 abrogated the depressive-like behavior induced by LPS (Wu et al., 2015).

Magnetic resonance imaging findings in the chronic mild stress (CMS) rodent animal, a model of depression, showed demyelination signs at the bilateral frontal cortex, hippocampus and hypothalamus, which revealed to be associated with brain oedema and inflammation (El-Etr et al., 2015). Gene expression profiling studies from patients with MDD showed that genes involved in energy metabolism and mitochondrial function were downregulated (Konradi et al., 2012), that genes involved in immune response and inflammation were upregulated (Shelton et al., 2011), and that genes expressed in oligodendrocytes were downregulated (Aston et al., 2005). These studies further corroborate the association between energy impairment, inflammation and myelin loss during MDD.

Non-responders to depressive treatment have shown increased baseline inflammation and oxidative stress (Strawbridge et al., 2015; Vaváková et al., 2015). A recent study evidenced that the antidepressant drug venlafaxine, though not having any influence on the majority of microglia-related proinflammatory parameters, significantly reduced superoxide production while revealed a protective effect on mitochondrial membrane potential, suggesting ability to prevent the progression of depression (Dubovický et al., 2014).

Inflammation-associated depression is now considered a clinical entity that provides an understanding on the dynamics of interaction between peripheral and brain mechanisms (Dantzer et al., 2011). Experimentally, acute immumostimulation by peripheral administration of LPS in mice caused sickness, increased immobility in the tail suspension test and depressive-like behavior in the forced swim test, together with a delayed cellular activity (Frenois et al., 2007; O’Connor et al., 2009). Some studies indicate that anti-inflammatory compounds in patients with inflammatory disorders and depressed patients may improve depressed mood, but conclusive data still waits confirmation (Miller et al., 2009; Song and Wang, 2011).

It is still a matter of debate whether a chronic inflammatory state may contribute to depression etiology or if inflammation occurs as a consequence of a depressive state. As mentioned above many stimuli related to depression may trigger microglia activation, namely: (i) peripheral or central inflammatory challenges; (ii) stress-related conditions derived from increase of glucocorticoids via HPA axis, reported to activate microglia (Sorrells and Sapolsky, 2007), from changes in gut microbiota, shown to control the maturation and functioning of microglia (Erny et al., 2015), or from psychological stress that promote microglial activation through the release of alarmins within the brain (Maslanik et al., 2013); and (iii) intense neuronal activity. This activation, may then promote the suppression of neurogenesis and neuroplasticity further enhancing the development of depression-like symptoms, suggesting that a prior inflammation may set the basis for the emergence of depression. Furthermore, continuous activation of microglia may concur to microglia function decline which have more recently been observed in depressive patients (Hannestad et al., 2013) and reported in several neurological conditions including aging, Alzheimer’s disease (AD), and chronic stress which are associated with a higher prevalence of MDD.

The study of cellular and molecular mechanisms of inflammation-associated depression will open new possibilities for developing new antidepressant compounds targeting neuroinflammation or its downstream pathways. Nevertheless, we should be cautious in believing that depression can be treated by therapies targeting inflammation. Further studies are required to evaluate whether a combined therapy with anti-inflammatory compounds and antidepressants will result in additional clinical benefits.

## Role of microRNAs in Depression

MiRNAs are a class of small noncoding RNAs that are key post-transcriptional regulators of gene expression, which may impair the translation of their target mRNA or promote its degradation, though they can on the contrary also act as translation activators or even impair transcription by binding to gene promoters (Krol et al., 2010; Younger and Corey, 2011). If we consider that more than 1400 miRNA genes were already identified, that each one may target a high number of different mRNAs, which individually can be suppressed by multiple mRNAs, there is no doubt on their role as robust determinants of cellular states (Krol et al., 2010; Rota et al., 2011; Lin and Gregory, 2015). Most of miRNAs are transcribed as pri-miRNAs by RNA polymerases II and III in the nucleus and cut into pre-miRNAs by the Drosha complex (Mouillet-Richard et al., 2012). Pre-miRNAs are transported to the cytoplasm by exportin 5 and Ran GTPase for final processing by the RNAse III enzyme Dicer generating 22 nt double-stranded mature miRNAs with a 5′ phosphate end (Gregory and Shiekhattar, 2005; Ksiazek-Winiarek et al., 2013). The mature miRNA is then incorporated into the RNA-induced silencing complex (RISC), which become able to repress translation (O’Connor et al., 2012).

Previous studies indicate that miR-124 and miR-128 are primarily expressed in neurons, whereas miR-23, miR-26, and miR-29 exist in large amount in astrocytes, supporting a differential nature of expression (Smirnova et al., 2005). Some miRNAs are associated with neurological functions such as learning and memory and miR-132, -134, and -let-7 are suggested to play a crucial role in the formation and plasticity of synapses, and miR-124a and miR-125b have been associated to the outgrowth of axons (Schratt et al., 2006; Le et al., 2009; see Table 1). MiR-124 is the most abundant in the brain and its dysregulation has been related with neurodegeneration, neuroimmune disorders and CNS stress among others (for review, see Sun et al., 2015). It was previously indicated to be a key regulator of adult neurogenesis (Cheng et al., 2009) and one of its targets is CREB (Rajasethupathy et al., 2009).

**Table 1:** **Critical and dysregulated microRNA (miR) in conditions of stress/depression**.

microRNA	Associated to depression-related pathways	Directly implicated in stress/depression	Reference
miR-1202	GRM4 as target. Modulator of glutamatergic, dopaminergic, GABAergic and serotonergic neurotransmission. Regulator of anxiety-related behaviors.	↓ in the MDD patient’s brain	Davis et al. (2012); Lopez et al. (2014) and Rucker and McGuffin (2014)
Let-7a	Neuronal differentiation of embryonic neural progenitors. Formation and plasticity of synapses.	↑ in the frontal cortex following acute stress	Schratt et al. (2006); Schwamborn et al. (2009) and Rinaldi et al. (2010)
miR-124	Important for neurogenesis.	↑ in the medial pre-frontal cortex following maternal separation stress	Uchida et al. (2010)
miR-29a	Targets Voltage Dependent Anion Channel and ATP synthetase.	↑ in the medial pre-frontal cortex following maternal separation stress	Uchida et al. (2010) and Bargaje et al. (2012)
miR-26a	Important in neuronal development and morphogenesis.	↑ in the frontal cortex following acute stress	Rinaldi et al. (2010) and Li and Sun (2013)
miR-26b	Induces cell cycle in postmitotic neurons and apoptosis.	↑ in the frontal cortex following acute stress	Rinaldi et al. (2010) and Absalon et al. (2013)
miR-26b, miR-1972, miR-4485, miR-4498, and miR-4743	Target biological processed involved in brain development and function: axon guidance and extension, synaptic transmission, learning and memory.	↑ in peripheral blood mononuclear cells from MDD patients	Fan et al. (2014)
miR-221-3p, miR-34a-5p, and let-7d-3p	Target serotonin receptors, corticotrophin-releasing hormone receptor and glutamate transporters. Enrich pathways related to neuronal function in depression.	↑ in serum from MDD patients	Wan et al. (2015)
miR-451a		↑ in serum from MDD patients
miR-132 and miR-182	BDNF as target.	↑ in serum from depressed patients. Polymorphism in the miR-182 gene is associated with MDD.	Li et al. (2013) and Saus et al. (2010)
miR-134	Can negatively regulate the size of dendritic spines.	↑ in amygdala after owing acute stress	Schratt et al. (2006) and Meerson et al. (2010)
miR-183	Regulates the circadian-clock period.	↑ in amygdala after owing acute stress	Xu et al. (2007) and Meerson et al. (2010)
miR-1302 and miR-625	P2XR7 as target. Neuronal receptor involved in synaptic transmission. Microglia scavenger receptor involved in phagocytosis.	SNP in putative miRNA target sites of miR-1302 and miR-625 of buccal epithelial cells from MDD patients	Sperlágh et al. (2006); Rahman et al. (2010) and Gu et al. (2011)
miR-9	Controls dendritic growth and synaptic transmission.	↑ in the frontal cortex following acute stress and in the medial pre-frontal cortex following maternal separation	Rinaldi et al. (2010); Uchida et al. (2010) and Giusti et al. (2014)
miR-144-5p	Targets PKC, Wnt/β-catenin, and PTEN pathways. Is involved in response to mood stabilizer treatment and stress responses.	↑ in the plasma of depressed patients	Zhou et al. (2009); Katsuura et al. (2012) and Wang et al. (2015)

BDNF, brain-derived neurotrophic factor; CSF, cerebrospinal fluid; GRM4, metabotropic glutamate receptor-4; PTEN, phosphatase and tensin homolog; PKC, protein kinase C; P2XR7, purinergic receptor P2x, ligand-gated ion channel 7; SNP, single nucleotide polymorphisms.

**Figure 1:**
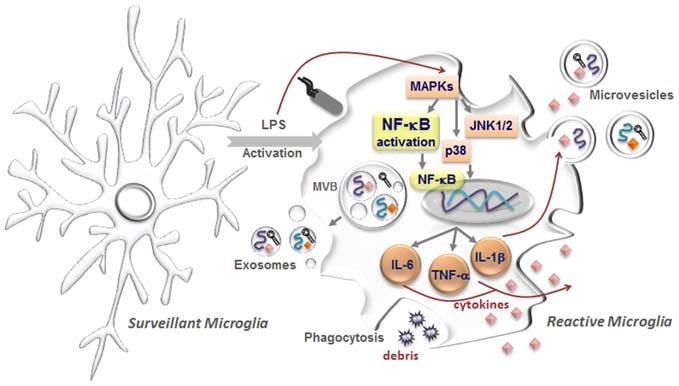
**Microglia activation with release of extracellular vesicles (EVs).** In the healthy central nervous system, microglia have highly ramified morphology with thin processes, which constantly monitor brain parenchyma to maintain the homeostasis. These microglia are commonly designated as surveillant microglia. Upon stimuli, namely by the proinflammatory lipopolysaccharide (LPS), reactive or activated microglia acquire different morphologies from hypertrophic with enlarged processes to an amoeboid shape. Intracellularly, several pathways become activated including the mitogen-activated protein kinases (MAPKs) superfamily, comprising the c-Jun N-terminal kinase (JNK 1/2) and p38 proteins, which will trigger the activation of the nuclear factor-κB (NF-κB) and consequent induction of first-line cytokine production, such as interleukin (IL)-1β, IL-6 and tumour necrosis factor (TNF)-α. In parallel, microglia are also able to sense cellular/molecular debris and intervene by phagocytosing them. Interestingly, messenger RNAs (mRNAs, curved symbols), microRNAs (miRNAs, black symbols) and cytokines (diamond-like symbols) are selectively incorporated into multivesicular bodies (MVBs) and may be released from activated microglia encapsulated in EVs, comprising exosomes and microvesicles. While exosomes are endocytic membrane-derived vesicles of small size (30–100 nm) that are contained in MVBs in the endosomal system and secreted upon MVB fusion with the plasma membrane, microvesicles/ectosomes are quite large vesicles (100–1000 nm) that bud directly from the plasma membrane. These vesicles will act as vehicles in cell-to-cell communication, being considered potential carriers of altered molecules promoting disease propagation.

Also involved in neurogenesis is the miR-137 associated with the development of neural stem cells into mature neurons (Szulwach et al., 2010). Synaptic plasticity is a critical process in learning and memory and its disruption may trigger psychiatric disorders. Recent evidence suggests that neuronal plasticity plays an important role in the recovery from depression and brain derived-neurotrophic factor (BDNF) is a mediator of this plasticity (Castrén and Rantamäki, 2010). Actually, BDNF was found decreased in depressed patients and in stressed animals (Dwivedi, 2009). In this context, miRNAs are known to influence BDNF, which may in turn induce the synthesis of miR-132 to regulate neurogenesis (Castrén and Rantamäki, 2010; Yan et al., 2013). The mitogen-activated protein kinases (MAPK) superfamily, including the extracellular signal-regulated kinase (ERK), c-Jun N-terminal kinase and p38 proteins, when activated in microglia trigger the release of pro-inflammatory mediators (Kaminska et al., 2009; see Figure 1), making MAPK signal transduction crucial in regulating gene expression and mediating a rapid response in stress-responsive microRNA expression (Biggar and Storey, 2011). Expression of miR-221 and miR-222 was shown to be related with ERK1/2 activation (Terasawa et al., 2009).

MiRNA expression was reported to be globally downregulated in the prefrontal cortex of depressed suicide victims (Smalheiser et al., 2012), which is consistent with the hypo-activation of the frontal cortex reported in depressed subjects (Covington et al., 2010). Those Authors observed that the 21 miRNAs found downregulated were related to cell growth and differentiation. However, miR-185 and miR-491-3p were increased in the frontal cortex of suicide completers (Serafini et al., 2014). Complementary studies using post-mortem human prefrontal cortex samples from MDD patients showed that miR-1202, a primate specific miRNA that is enriched in the human brain, was down-regulated in depressed individuals (Lopez et al., 2014). This miR-1202 targets the expression of the gene encoding metabotropic glutamate receptor-4 (GRM4), which was also up-regulated in MDD patient samples. This receptor is localized pre- and post-synaptically and functions as modulator of glutamatergic, dopaminergic, GABAergic and serotonergic neurotransmission (Pilc et al., 2008). More recently, it has been pointed as a regulator of anxiety-related behaviors (Davis et al., 2012).

A different study analyzed miRNAs in the peripheral blood mononuclear cells (PBMCs) of MDD patient and identified that 5 miRNAs were up-regulated, such as miRNA-26b, miRNA-1972, miRNA-4485, miRNA-4498, and miRNA-4743 (Fan et al., 2014; see Table 1). The Authors next looked at predicted targets of these miRNAs and identified genes with a wide variety of biological effects, including axon guidance and extension, synaptic transmission, learning and memory, which changes have been associated to the pathophysiology of MDD. In addition, another work evidenced that the plasma expression of miR-144-5p was inversely associated with depression and suggested its origin from the pathologic processes of depression (Wang et al., 2015). MiR-144-5p is involved in the response to mood stabilizer treatment (Zhou et al., 2009) and stress responses (Katsuura et al., 2012), and target the protein kinase C (PKC), Wnt/β-catenin, and phosphatase and tensin homolog (PTEN) pathways (Zhou et al., 2009).

Several altered miRNAs were also identified in the CSF of MDD patients but, notably, elevated miR-221-3p, miR-34a-5p and let-7d-3p, together with low miR-451a levels in serum, were suggested as potential biomarkers of MDD (Wan et al., 2015). Among the 16 miRNAs identified part of them were reported to be down-regulated during antidepressant medications (miR-30a-5p, miR-34a-5p and miR-221; Zhou et al., 2009). Analysis of their target genes showed that some important genes related to MDD, such as serotonin receptors (HTR2C), corticotrophin-releasing hormone receptor (CRHR1) and glutamate transporters (SCL1A2), were down-regulated. In contrast, diverse pathways linked to neuronal function in depression, including axon guidance, Wnt signaling pathway, neurotrophin signaling pathway, and long-term depression, were associated to alterations in miR-451a, let-7d-3p, miR-221-3p and miR-34a-5p in both serum and CSF. This study highlighted the existence of differentially altered miRNAs in CSF and serum of MDD patients and their modulation by antidepressant treatment suggesting their potential use as biomarkers for MDD. Curiously, overexpression of miR-34a, miR-30a-5p and let-7d were also shown to down-regulate BDNF expression (Croce et al., 2013).

Data from a mouse model with learned helplessness—an analog for depressive symptoms, demonstrated up-regulation of a polycistronic miRNA cluster (including miR-96, miR-182, and miR-183) that was shown to target genes in step with the circadian clock (Xu et al., 2007). In accordance, increased serum levels of miR-182 and also miR-132 were also found in patients with depression (Xu et al., 2007). Most attractive was the report that both miRNAs targeted BDNF, which have also been detected at lower serum levels in patients with depression (Pallavi et al., 2013). Recently, it was shown that genetic variations in microRNA processing genes, namely *DGR8* and *AG01* (variant AGOl rs636832), were associated with depression (He et al., 2012). Therefore, given the role of miRNAs as potential regulators of neurogenesis and neural plasticity, and the studies suggesting that miRNA processing polymorphisms may contribute to depression risk and response to treatment (Dwivedi, 2014), it is anticipated that miRNAs may constitute a diagnostic tool as well as targets to novel medicines.

## Exosomes and Ectosomes as Mediators of Neuroinflammation

All neural cells, including neurons, astrocytes, oligodendrocytes and microglia, release EVs comprising exosomes and ectosomes, either in normal or pathological conditions (Gupta and Pulliam, 2014). For instance exosomes and ectosomes were shown to be implicated in amyloid-beta (Aβ) cell-to-cell spreading and neurotoxicity (Brites, 2015). In addition, proteins recruited to exosomes were suggested to be linked to overexpression of tau and associated to both toxicity and neurofibrillary lesion spreading in AD (Saman et al., 2014). Lately, it was suggested that increased incorporation of P-S396-tau, P-T181-tau, and Aβ1–42 in neurally derived blood exosomes predict the occurrence of AD up to 10 years before clinical onset (Fiandaca et al., 2015). Most curious tau dysfunction, in addition to the direct effects of Aβ, may drive alterations in microglial phenotypes and neuroinflammation (Metcalfe and Figueiredo-Pereira, 2010; Wes et al., 2014). This may justify why minocycline, an anti-inflammatory agent, was shown to reduce neuroinflammation and to restore cognition in an AD mouse model (Parachikova et al., 2010).

Transfer of toxic proteins by exosomes include α-synuclein with a concomitant increase in recipient cells factors that is associated with Parkinson’s disease (PD) pathology and progression, thus providing a suitable target for therapeutic intervention (Alvarez-Erviti et al., 2011a; Bellingham et al., 2012b). Moreover, it was demonstrated that α-synuclein can induce an increase of microglia secreted exosomes containing a high level of major histocompatibility complex (MHCs) class II molecules and membrane TNF-α (Chang et al., 2013). Exosomes derived from astrocytes and motor neurons also showed a key role in the amyotrophic lateral sclerosis (ALS) disease based on studies demonstrating the efficient transfer of mutant and misfolded copper-zinc superoxide dismutase 1 (SOD1) to other cells, reason why exosomes are now suggested as targets to modulate ALS disease (Basso et al., 2013; Grad et al., 2014).

Aberrantly expressed cellular miRNAs, selectively packaged and transported in exosomes, can lead to dysregulated gene expression in the recipient cell (Gupta and Pulliam, 2014). Exosomes released by prion-infected neuronal cells showed increased let-7b, let-7i, miR-128a, miR-21, miR-222, miR-29b, miR-342-3p and miR-424 levels, together with reduced miR-146a levels as compared to non-infected exosomes (Bellingham et al., 2012a). Exosomes were associated to the pathogenesis of infectious CNS diseases, prion disease, AD, PD and ALS (Brites and Vaz, 2014; Gupta and Pulliam, 2014; Brites, 2015).

## Biogenesis of Exosomes and Ectosomes

Cells release into the extracellular environment several types of membrane vesicles from endosomal and plasma membrane origin designated by exosomes and MVs or ectosomes, respectively (Raposo and Stoorvogel, 2013). These EVs, a designation that was recommended for the two classes of vesicles (Cocucci and Meldolesi, 2015), represent an important mode of intercellular communication by serving as vehicles for transfer and delivery of membrane and cytosolic proteins, lipids, mRNAs, and microRNAs (miRNAs) between cells (Lin et al., 2015). EVs are present in many if not all bodily fluids, including blood, urine, saliva, amniotic fluid, breast milk and culture medium of cell cultures (Théry et al., 2002, 2006). Since they contain biologically active proteins and regulatory RNAs, they are suggested to be associated with the propagation of a disease and to create a microenvironment that may favor disease progression (Vingtdeux et al., 2012; Kahlert and Kalluri, 2013).

EVs also include other denominations besides exosomes and ectosomes, as shedding vesicles, nanoparticles, microparticles and oncomes among others (Cocucci and Meldolesi, 2015). Exosomes are endocytic membrane-derived vesicles of small size (30–100 nm) that are contained in multivesicular bodies (MVBs) in the endosomal system and secreted upon MVB fusion with the plasma membrane (Prada et al., 2013; see Figure 1). MVs observed by transmission electron microscopy pictures exhibit characteristic cup-shaped or ellipsoid morphology (Momen-Heravi et al., 2012; Fertig et al., 2014). Secretion of exosomes derived from motor neuron-like NSC-34 cells overexpressing mutant hSOD1G93A was proposed as a mechanism of cell-to-cell transfer of mutant SOD1 toxicity (Gomes et al., 2007). These cells indeed release with the characteristic cup-shape morphology by transmission electron microscopy.

Although little is known about MVB fusion with the plasma membrane, several Rab GTPases, including Rab5, Rab27, and Rab35, were suggested to be involved (Shifrin et al., 2013). Exosomal membranes are enriched in cholesterol and sphingomyelin and certain membrane proteins, such as tetraspanins and integrins (Frühbeis et al., 2012). Exosome production may be inhibited by targeting neutral sphingomyelinase-2 with GW4869 (Yuyama et al., 2012) or manumycin-A that also showed to impair transfer of miRNAs to other cells (Mittelbrunn et al., 2011). MVs/ectosomes are quite large vesicles (100–1000 nm) that bud directly from the plasma membrane (Turola et al., 2012; see Figure 1). The rate of ectosome shedding is variable, with cells showing elevated formation and release, and others a low rate of ectosome formation (Cocucci and Meldolesi, 2011). Although there are differences on the biogenesis of exosomes and ectosomes, the two EVs display a similar function when released (Cocucci and Meldolesi, 2015). CD63 and CD61 are indicated as markers of exosomes and TyA and C1q of ectosomes, considering the uncertainty of many others that have been appointed. The time of release varies from delayed in exosomes to seconds in ectosomes. Moreover, only 5% of exosomes have externalized phosphatidylserine while MVs/ectosomes evidence a major representation (Prada et al., 2013). The removal of plasma membrane by ectocytosis is compensated by the fusion of MVBs as a compensatory exocytic process of preformed intracellular vesicles (Sadallah et al., 2011; Cocucci and Meldolesi, 2015).

## Role of Vesicles in Neuron—Glia Communication

Brain function depends on coordinated interactions between neurons and glial cells that include microglia, astrocytes, and oligodendrocytes. These cells release vesicles in a way regulated by glutamate (Chivet et al., 2014), which participate in the communication between those cells. EVs released from primary cortical astrocytes and microglial cells appear to be triggered by ATP-mediated activation of P2X7 receptors, after exposure of phosphatidylserine at the cell surface, followed by downstream stimulation of acid sphingomyelinase (Bianco et al., 2009).

Immature and reactive astrocytes in primary cultures were shown to release large vesicles (up to 8 μm) from the cell surface containing functional mitochondria and lipid droplets, probably as a consequence of repetitive ATP stimulation (Falchi et al., 2013). Vesicle delivery to the plasma membrane involves interaction with the cytoskeletal microtubules and actin filaments (Kreft et al., 2009). Astrocytes also release smaller EVs with approximately 100 nm in size (exosomes; Gosselin et al., 2013) containing both neuroprotective and neurotoxic molecules. Astrocyte-derived MVs and exosomes were described to present neuroprotective proteins including synapsin 1 (Wang et al., 2011), molecules implicated in angiogenesis such as VEGF (Proia et al., 2008), and matrix metalloproteinases involved in extracellular matrix proteolysis (Sbai et al., 2010). Stressful events in astrocytes, like heat or oxidative stress, were shown to induce the release of exosomes carrying the heat shock protein 70 (Hsp70) and synapsin 1 with a pro-survival effect on neurons (Taylor et al., 2007). Upon pathological conditions it has been suggested a detrimental role of astrocyte-derived EVs in the propagation of pathogenic proteins in the course of neurodegenerative disorders. Astrocytes expressing SOD1 mutant, involved in familiar ALS, release a higher amount of exosomes carrying this mutant protein, which were shown to promote *in vitro* motor neuron death following SOD1 efficient transfer (Basso et al., 2013). Further, astrocytes exposed to Aβ species evidenced to secrete pro-apoptotic exosomes which may be taken up by astrocytes themselves or by other neighboring cells contributing to AD neurodegeneration (Wang et al., 2012). Most attractive, it was recently showed that the functional astrocytic excitatory amino-acid transporter (EAAT)-1 is present in secreted EVs, increasing its concentration upon astrocyte activation (Gosselin et al., 2013), suggesting a possible role of this microvesicular transporters in reducing excitotoxicity at an extracellular level.

Oligodendrocyte-axon interaction constitutes a functional unit allowing neuronal integrity (Nave, 2010). Oligodendrocytes secrete exosomes in a calcium-dependent fashion containing myelin proteins and lipids (Frühbeis et al., 2012). These exosomes seem to convey autocrine signals (Bakhti et al., 2011), but can also be internalized by microglia by micropinocytosis to be degraded, thus avoiding an immune reaction (Fitzner et al., 2011). Early studies suggest that oligodendrocyte exosomes may play a crucial role in allowing the supply of myelin proteins (i.e., proteolipid protein and myelin oligodendrocyte glycoprotein), heat-shock proteins, glycolytic enzymes and more recently glycolytic substrates including lactate (Krämer-Albers et al., 2007; Lee et al., 2012). Oligodendroglial exosomes may be also internalized by neurons through endocytosis and their cargo may contribute to neuroprotection and long-term axonal maintenance (Frühbeis et al., 2012). Moreover, the release of oligodendrocyte-derived exosomes seems to be modulated through neuronal signaling (Frühbeis et al., 2013). Glutamate release from neurons activates oligodendrocytes through N-methyl-D-aspartate receptor (NMDA) engagement leading to an increase of intracellular calcium and exosome secretion. These exosomes are then internalized specifically by neurons, and increased firing rate and altered gene expression upon oligodendroglial exosome exposure was observed (Frohlich et al., 2014), confirming that oligodendrocytes influence neuronal physiology, either by induction of signaling cascades or by transfer of mRNAs and miRNAs. Oligodendrocyte-derived MVs have also been described to inhibit both the morphological differentiation of oligodendrocytes and myelin formation through neuronal modulation (Bakhti et al., 2011), suggesting a sophisticated control of myelin membrane biogenesis via MVs. As a physiological degradation of oligodendroglial membrane, part of oligodendrocyte-derived exosomes may be internalized by MHC class II negative microglia through macropinocytosis being subsequently cleared via the lysosomal pathway with no inflammatory response (Fitzner et al., 2011). However, in pathological situations such as AD, where activated microglia are associated with an intense inflammatory milieu, oligodendrocytes were reported to secrete EVs containing degraded myelin proteins (Zhan et al., 2014). Upon stressful conditions, oligodendrocytes may also release immune mediators and express known chemoattractants, signifying an active role on microglia activation and recruitment to the injured area (Peferoen et al., 2014). Microglia activation and MV release will be the subject of the next section.

Recent evidences indicate that exosomes from neuroblastoma cells bind to neurons and glial cells, despite being preferentially endocytosed by glia, while those released upon synaptic activation bind selectively to other neurons (Chivet et al., 2014). On the other hand, the release of serotonin from neurons was evidenced to regulate microglia-derived exosomes involving the elevation of intracellular calcium, at least under physiological conditions, what suggests a neurotransmitter dependent release (Glebov et al., 2015). Most curious, it was also demonstrated that exosomes secreted by Schwann cells, the peripheral glial cell type, are internalized by neurons increasing neurite growth substantially and axonal regeneration (Lopez-Verrilli et al., 2013). Most relevant, intercellular chaperone transmission mediated by exosomes was demonstrated to contribute to maintenance of protein homeostasis (proteostasis; Takeuchi et al., 2015). Moreover, the secretion of harmful or unwanted material in exosomes together with the autophagy-lysosomal pathway also contribute to preserve intracellular protein and RNA homeostasis (Baixauli et al., 2014).

Besides the biological roles of EVs in the CNS, these EVs are also correlated with several neurological diseases, as already indicated. To be noted that alterations in exosome composition in some pathological conditions may switch the immunologically inert exosomes into active ones triggering inflammatory reactions in the CNS. EVs from oligodendroglioma cells revealed to induce astrocyte cell death in primary cultures, which was suggested to be mediated by the pro-apoptotic effects of Fas ligand (FAS-L) and TNF-related apoptosis-inducing ligand (TRAIL; Lo Cicero et al., 2011). Moreover, it was mentioned that exosome-associated amyloids can act as seeds for plaque formation contributing to AD (Rajendran et al., 2014). Intriguingly, the transfer of miR-1 in EVs from the glioblastoma cells was shown to induce multiple changes in the glioblastoma multiform (GBM) surrounding cells (Bronisz et al., 2014). Involvement of exosomes in pathological processes is also associated with their role as potential carriers of misfolded proteins (Bellingham et al., 2012b; Russo et al., 2012; Schneider and Simons, 2013). Cell-to-cell communication by exosomes has been related to the PD progression. Neuronal exosomes containing α-synuclein were suggested to be transmitted from neuron-to-neuron and from neuron-to-glia, while those from activated glial cells to be transferred in a glia-to-glia process, leading to disease spread and propagation of the inflammatory response, respectively (Russo et al., 2012).

## Exosomal microRNA Signature

Cumulating data suggest that miRNAs are potential biomarkers for the diagnosis and prognosis of a variety of diseases such as cancer and neurological diseases (Mishra, 2014; Warnecke-Eberz et al., 2015). Although no references associate exosomes with depression syndromes, several reports highlight the involvement of miRNAs and posttranscriptional dysregulation in psychiatric disorders (see Table 1). One of the first association of depression with altered miRNAs was described for P2RX7 (purinergic receptor P2x, ligand-gated ion channel 7) gene, where single nucleotide polymorphisms (SNPs) were identified in putative miRNA target sites of miR-1302 and miR-625 within the P2RX7 3′-untranslated region (Rahman et al., 2010). P2RX7 channel is involved in synaptic neurotransmitter regulation (Sperlágh et al., 2006) and is present in microglia acting as a scavenger receptor in the absence of its ligand ATP, mediating phagocytosis of apoptotic cells and insoluble debris (Gu et al., 2011).

**Figure 2:**
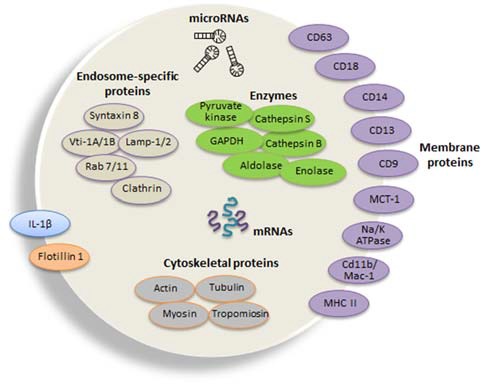
**Composition of typical microglial exosomes.** Exosomes are packed with several cellular components including messenger RNAs (mRNAs, curved symbols), microRNAs (miRNAs, black symbols) and proteins. Microglia-derived exosomes express specific markers of late endosomes corroborating their organelle origin, as well as major histocompatibility complexes (MHCs) class II molecules and enzymes (i.e., cathepsin S) indicative of their function as antigen presenting cells, and integrins involved in antigen presentation and pattern recognition receptors important for innate immunity. Flotillin 1 is a membrane-associated protein that is enriched in exosomes and thus commonly used as an exosomal marker. Tetraspanins are a family of transmembrane proteins that become integrated in the membrane of EVs and among them are CD9, CD63 and CD81, which are particularly enriched in microglia-derived exosome. The surface-bound aminopeptidase CD13 that degrades enkephalins and the lactate transporter monocarboxylate transporter 1MCT-1 are also highly expressed in exosomes released from microglial cells. CD14 is a monocyte/macrophage marker, regularly used to characterize exosomal preparations. Extrusion of Na, K-ATPase is likewise frequent in different populations of exosomes. Microvesicles also carry the proinflammatory cytokine interleukin (IL-1β) that is shed from the plasma membrane of the microglial cell upon ATP stimulation. In addition, exosomes may additionally contain a distinct set of proteins, such as cytoskeletal proteins and the glycolytic protein glyceraldehyde 3-phosphate dehydrogenase (GAPDH). The illustration is based on proteomic analysis of exosome preparations derived from N9 microglial cells (Potolicchio et al., 2005).

Recently, mRNAs and miRNAs were identified in exosomes and showed to derive from an active sorting mechanism since the miRNA profiling revealed that it may differ from that of the cells of origin (Zhang et al., 2015). The latest exosome content database identified 4563 proteins, 194 lipids, 1639 mRNAs, and 764 miRNAs in exosomes from multiple organisms (Mathivanan et al., 2012). Electron microscopic analysis revealed that microglia release exosomes with proteins already identified in B cell- and dendritic cell (DC)3-derived exosomes, but with unique proteins such as the aminopeptidase CD13 and the lactate transporter MCT1, which may supply supplementary energy substrate to neurons during synaptic activity (Potolicchio et al., 2005; see Figure 2 for the typical protein composition of exosomes derived from microglia). The Authors used the N9 microglial cell line and analysis by mass spectroscopic peptide mapping, Western blotting, and enzymatic analysis. They found that N9-derived exosomes express specific markers of late endosomes corroborating their organelle origin, as well as MHC class II molecules and cathepsin S indicative of their function as antigen presenting cells. Integrins involved in antigen presentation and pattern recognition receptors important for innate immunity were also detected.

Recent evidence suggests that miRNAs are transferred by exosomes to recipient cells, while altering gene expression and mediating functional properties (Valadi et al., 2007; Mittelbrunn et al., 2011; Turchinovich et al., 2013; Weber, 2013). Exosomal miRNA was indicated to be released via ceramide-dependent secretory pathways controled by neutral sphingomyelinase that is crucial for budding of intracellular vesicles into MVBs (Trajkovic et al., 2008; Kosaka et al., 2010b; Kogure et al., 2011; Mittelbrunn et al., 2011). However, the precise mechanisms of vesicular miRNAs sorting and secretion are still to be clarified.

Since the majority of current reports describing isolation of MVs-associated extracellular miRNA only rely on ultracentrifugation, one should understand that such experiments inevitably characterize miRNAs in a mixed population of MVs and exosomes (Turchinovich et al., 2013). This is the case of a recent study identifying the presence of miR-155 and miR-146a, two critical inflammation-related miRNAs that showed to modulate microglia phenotype (Cardoso et al., 2015), in exosomes that are released from and taken up by dendritic cells (Alexander et al., 2015). The Authors demonstrated that injection of miR-146a-containig exosomes inhibited, while that of miR-155 promoted, the endotoxin-induced inflammation in mice. Moreover, they showed that one dendritic cell produces near 500 exosomes after 24 h of culture and that each one contains about one copy of miR-146a.

It is presently considered that there are four main processes for sorting miRNAs into exosomes: (i) The neural sphingomyelinase 2-dependent pathway; (ii) The miRNA motif and sumoylated heterogeneous nuclear ribonucleoproteins-dependent pathway; (iii) The 3′-end of the miRNA sequence-dependent pathway; and (iv) The miRNA induced silencing complex-related pathway (Zhang et al., 2015). It should be considered that besides the involvement of specific sequences in certain miRNAs in guiding their incorporation into exosomes, sorting of exosomal miRNAs may be processed under the control of enzymes or other proteins in a miRNA sequence-independent manner (Khalyfa and Gozal, 2014).

Exosomal miRNAs have been indicated as a promising platform for compartment biomarker and early detection of diseases, including brain disorders (Cheng et al., 2014; Khalyfa and Gozal, 2014; Hornick et al., 2015; Lin et al., 2015; Schwarzenbach, 2015). Recent advances in the research of exosomal biomarkers and their potential application in clinical diagnostics will be addressed in the last section of this review.

## Microglia Activation and Microvesicle Release

Emerging data indicate that activation of microglia is associated to both secretion of soluble molecules and release of EVs into the pericellular space. In the healthy CNS, microglia have highly ramified morphology with thin processes, which dynamically monitor the neural cell microenvironment for surveillance in efforts to maintain homeostasis (Nimmerjahn et al., 2005; Kettenmann et al., 2011; see Figure 1). Reactive or activated microglia acquire several altered morphologies, including a hypertrophic cell with enlarged processes, or an amoeboid shape (reviewed in Gemma and Bachstetter, 2013). Depending on the stimuli and on the extent of activation, microglia acquire several phenotypes, being the M1 (inflammatory cell) and M2 subtypes (pro-regenerative cells) the most commonly indicated (Brites and Vaz, 2014).

The proinflammatory phenotype intends to protect and repair the CNS from being damaged (Czeh et al., 2011; Chhor et al., 2013; Brites and Vaz, 2014). However, excessive and prolonged neuroinflammation can be cytotoxic and harmful (for review, see Cherry et al., 2014). The M2 phenotype, or the alternatively activated state, inhibits inflammation and works to restore homeostasis. This phenotype may include different subtypes: (i) M2a associated with the production of anti-inflammatory cytokines and trophic factors (Colton, 2009); (ii) M2b considered to be a combined M1/M2a subtypes (Brites et al., 2015); and (iii) M2c associated with phagocytosis and suppression of the innate immune system (Brites and Vaz, 2014). However, one should be cautious when considering that M2 microglia is always advantageous, once it was suggested to correspond to a deactivated, irresponsive population (Chakrabarty et al., 2012), and M2a together with M2c cells were shown to be implicated in AD progression in the APP/PS1 transgenic mice (Weekman et al., 2014). Actually, the notion of microglia as either “good” or “bad” is believed to be too simplistic because microglia switch between these phenotypes and may exist in many intermediate states. Today it is also believed that surveillant microglia is able to phagocytose spines and apoptotic cells (Sierra et al., 2013). Further studies are required to decipher the participation of microglia subtypes in cell homeostasis and neuroinflammation under normal and pathological circumstances.

Non-vesicular mechanisms of microglial secretion mediated by connexin (Cx)43, Cx36, and P2X7 ATP channels are increased in M1 microglia (Eugenín et al., 2003; Dobrenis et al., 2005; Choi et al., 2007). Recent data have shown that reactive microglia release EVs, which are implicated in communication with the brain microenvironment (for review, see Prada et al., 2013). MVs, but not exosomes, seem to contain the cytokine IL-1β and its proform, together with pro-caspase-1, the inflammasome-associated enzyme responsible for IL-1β maturation, when microglia is stimulated by LPS (Bianco et al., 2005). Most interesting, MVs derived from either M1 or surveillant microglia were shown to modulate synaptic activity (Prada et al., 2013). Recent studies evidenced that active synapses promote the pruning of inactive ones by stimulating microglial phagocytosis with exosomes (Bahrini et al., 2015).

Through these processes, microglia influence brain cell functions, either by propagating inflammation and causing neurodegeneration or by playing a neuroprotective role. Indeed, it was demonstrated that MVs from reactive microglia induce an inflammatory reaction in target cells (Verderio et al., 2012a) and that α-synuclein treated microglia release activated exosomes, triggering increased apoptosis and with a suggestive role in the progression of PD (Chang et al., 2013). Microglia-derived MVs were also shown to participate in AD neurodegeneration by promoting the formation of soluble Aβ species and the propagation of such toxic forms (Joshi et al., 2014). On the other way, it was demonstrated that interferon (IFN)-γ stimulated microglia release nutritive exosomes that conferred protection to the neighboring cells (Pusic and Kraig, 2015). The release of MVs, at least from macrophages, was shown to be enhanced when cells are treated with ATP and when polarized either in M1 or in M2 phenotypes. The content of such MVS seems to be determined by the polarization state in that nucleic acid content was shown to be specific (Garzetti et al., 2013). Moreover, different stimuli may trigger the release of MVs with distinct properties, as observed with monocytes (Bernimoulin et al., 2009). Some studies also evidence that induced and proliferative senescence is associated with increased release of MVs, mainly exosomes (Lehmann et al., 2008). Actually, exosome production and secretion are altered during *in vivo* aging and in cancer, and their cargo in miRNAs was suggested to contribute to aging (Xu and Tahara, 2013). One may then assume that activation lead to an increased release of MVs that may differ on their cargo and be associated with several physiological and pathological processes in which neuroinflammation play a pivotal role. Moreover, microglial decline and senescence was suggested to also be implicated in depression-associated impairments such as neuroplasticity and neurogenesis (Yirmiya et al., 2015). Recently, it was demonstrated two principal mechanisms of transmembrane protein release from senescent cells, one related with tumour necrosis factor receptor 1 (TNFR1) by ectodomain shedding and the other of the full-length intercellular adhesion molecule 1 (ICAM1) through MVs, probably exosomes (Effenberger et al., 2014). Interestingly while autophagy was shown to decrease with age, the release of exosomes seems to be enhanced in senescent cells (Brites, 2015).

## Extracellular Vesicles in Advanced Medicine

EVs have been investigated in neurodegenerative diseases as potential biomarkers, improving diagnosis and disease monitoring, as well as vehicles for targeted delivery of pharmacological compounds and gene therapies. Most attractive is that each EV relies on its source cell. They share expression level, presence, absence, mutation, copy number variation, truncation, duplication, insertion, modification, sequence variation, or molecular association of a given molecule being defined as a bio-signature. According with this concept of EVs bio-signature, analysis of EVs in circulating fluids may allow the identification of their cellular source and constitute important disease biomarkers, either in diagnosis or prognosis.

It has been proposed that serotonin dysfunctions are implicated in the pathophysiology of MDD and decreased levels were found in MDD patients (Paul-Savoie et al., 2011). Thus, serotonin remains at the core of recent advances to elucidate the underlying mechanisms of depression (Albert et al., 2012). Most interesting serotonin was demonstrated to stimulate the secretion of exosomes from microglia cells (Glebov et al., 2015). We may speculate that low levels of serotonin in MDD patients may influence the microglia release of exosomes and be related with the pathology. Indeed, decreased exosome formation was demonstrated to trigger Aβ aggregation for instance, reason why exosome administration or enhancement of exosome generation was suggested as a novel therapeutic approach to AD (Yuyama et al., 2014). In the future, development of engineered nanovesicles might be a valuable tool for the therapy of pathologies associated with inflammation and oxidative stress, including MDD. Future work on the role of exosomes in MDD is required.

As previously noted exosomes may be used as delivery platforms, encapsulating agents or siRNAs, but also as a diagnostic tool due to their content in miRNAs and misfolded proteins. The potential of miRNAs to serve as biomarkers in a noninvasive manner for psychiatric and neurodegenerative diseases and to monitor antidepressant response has been progressing (Dwivedi, 2011, 2014; Serafini et al., 2012; Chana et al., 2013; Dorval et al., 2013). Based on studies demonstrating the role of miRNAs in neural plasticity, neurogenesis and stress response, it is believed that they may contribute to the pathogenesis and progression of MDD. For example, acute stress and chronic stress induce an increase in the expression of selected miRNAs, including miR-134, miR-183, miR-132, Let-7a-l, miR-9–1, and miR-124a-l in the brain (for review, see Dwivedi, 2014). Dysregulated miRNAs were observed in PBMCs from patients with MDD and consistent overexpression along 8-weeks interval was obtained for miR-941 and miR-589 (Belzeaux et al., 2012). As already mentioned, actively secreted miRNAs are enclosed in exosomes, which are excreted in response to stress signaling (Mendell and Olson, 2012) and thus considered as potential biomarkers in depression and antidepressant response (Dwivedi, 2014).

Exosomal miRNAs may constitute biomarkers of a specific disease, as their serum levels are altered in a variety of pathological conditions (Kosaka et al., 2010a; Etheridge et al., 2011; Mo et al., 2012). Indeed, serum enrichment of exosomal miR-21 was positively correlated with tumour progression and aggressiveness in patients with oesophageal squamous cell carcinoma (Tanaka et al., 2013), miR-19a with the recurrence in human colorectal cancer (Matsumura et al., 2015) and miR-141 with metastatic prostate cancer (Kim and Kim, 2013). Increased levels of serum exosomal miR-150, -155, miR-342 and -1246 may differentiate patients with acute myeloid leukemia (Fayyad-Kazan et al., 2013; Hornick et al., 2015) and miR-122 can be an indicator of liver injury and portal hypertension (Jansen et al., 2015).

Brain exosomal miRNAs were shown to distinguish patients with schizophrenia (increased expression of miR-497) from those with bipolar disorder (increased expression of miR-29c; Banigan et al., 2013). Brain-derived EVs can travel and be detected in CSF (Verderio et al., 2012b) and based on the fact that they cross the blood-brain barrier (BBB) from the periphery into the brain (Gupta and Pulliam, 2014) we may hypothesize that the opposite is also true. Therefore, a significant body of literature indicate exosomes as novel biomarkers in clinical diagnosis with high specificity and sensitivity derived from their excellent stability, although their potential value in clinical diagnostics still needs to be fully explored. Recent studies in PD provided evidence on the existence of a circulating subpopulation of MVs showing exosomal properties and enriched Integrin β1 content (Tomlinson et al., 2015).

Nowadays it is considered that by engineering microglia it will be possible to modulate derived EVs and redirect microglia towards a neuroprotective phenotype able to promote tissue repair and with promising therapeutic effects in neurodegenerative diseases associated to inflammatory processes. In particular, short interfering RNAs (siRNAs) are now recognized as therapeutic tools that despite their poor bioavailability can cross the BBB when administered by systemic injection of target exosomes (Alvarez-Erviti et al., 2011b; El Andaloussi et al., 2013). When purified exosomes were loaded with exogenous siRNA by electroporation, strong mRNA and BACE1 protein knockdown, a therapeutic target in AD, was observed in wild-type mice (Alvarez-Erviti et al., 2011b). This was the first demonstration of an exosome-based drug delivery system. Furthermore, intracerebrally administered exosomes revealed to act as potent scavengers for Aβ by carrying it on the exosome surface, thus improving Aβ clearance what may have potential benefits in AD (Yuyama et al., 2014). It was additionally demonstrated that exosomes are able to transfer siRNA to monocytes and lymphocytes triggering the silencing of the target gene *MAPK* (Wahlgren et al., 2012) or knocking down the target gene *RAD51* in recipient cancer cells (Shtam et al., 2013). It was shown that exosomes can also be loaded with interference RNA (iRNA) and most curious, when injected intravenously in mice they were identified only in the target cells (Alvarez-Erviti et al., 2011b).

There is a growing interest in exploring exosomes and their cargo as a tool to monitor disease and as having therapeutic potential as delivery vehicles for specific miRNAs and/or their inhibitors (Kim et al., 2006; Bhatnagar et al., 2007; Lai et al., 2011; Yang et al., 2011; Hu et al., 2012; Ohno et al., 2013). The modulation of miRNA functioning may be achieved through overexpression or by knocking down in EVs. As an example, transfer of anti-miR-9 from mesenchymal stem cells (MSCs) into GBM cells was able to reverse the chemoresistance by blocking the expression of P-glycoprotein in GMB cells and was mediated by the release of MVs (Munoz et al., 2013). Exosomes are considered ideal for drug delivery due to the low immunogenicity, capacity to transport molecules, ability to interact with target cells and willingness to be manipulated for personalized medicine (Gupta and Pulliam, 2014). Such approach is of particular significance once the utilization of miRNAs as therapeutics using a systemic approach needs to overcome the gastrointestinal system, cross the BBB and produce the desired effect in a specific part of the brain (O’Connor et al., 2012).

There is a large body of information showing that soluble factors and EVs within the secretome provide a major contribution to paracrine activity generating a tissue microenvironment that may be neurotoxic or beneficial to regeneration. Secretome from NSCs and MSCs was shown to contain NGF, glial derived neurotrophic factor (GDNF), and BDNF, among others, and suggested to modulate the neurogenic niche (Salgado et al., 2015). As the neurogenic process in the adult brain constitutes a new dimension of plasticity, ways to repair impairments in neuroplasticity may turn useful to treat depression (Bessa et al., 2009; Mateus-Pinheiro et al., 2013a,b). Other studies provided data showing that MSCs and their secretome are able to rescue the AD cell model from misfolded truncated tau-induced cell death (Zilka et al., 2011). Therefore, another therapeutic option will be to inject the stem cell secretome for repair of the damaged tissue, which may even be improved through the use of gene expression methodologies or culture preconditioning of modified stem cells increasing the ability to secrete pro-regenerative factors (Drago et al., 2013).

EVs may be produced from stem cells and their cargo modified to be enriched in growth factors, cytokines, chemokines and regulatory miRNAs to achieve a faster and better regeneration of the injured tissue (Ratajczak et al., 2012; Drago et al., 2013). Most encouraging, EVs can be generated from patient’s cells and used for autologous therapies after modulation of their miRNA cargo. Although requiring a great deal of future studies to understand how miRNAs are transferred into exosomes and delivered to target cells, it is expected that exosomes and/or ectosomes will be soon used in clinics. Considering the link between neuroinflammation and depression, we may assume that circulating exosomes from activated glia may contain increased levels of MDD-related miRNAs, which if modulated can switch exosomal neurotoxic effects to neuroprotective activities. This is a field not yet explored in MDD that deserves to be investigated in the future inasmuch pathophysiology leading to mood disorders may differ among patients.

## Future Perspectives

MDD is a disabling condition with impact on well-being and a leading cause of disease burden in high-income countries. Treatment of MDD is very challenging since some patients do not respond to first-line pharmacotherapy. Current guidelines for the management of MDD recommend selective serotonin receptor inhibitors or serotonin-norepinephrine receptor inhibitors, but due to side effects, including potential increased suicidal ideation, clinicians are considering atypical antipsychotics as alternative therapies. Augmentation of MDD patients that do not respond adequately to antipsychotics has increased over the past decade. Whether exosomes may help in the treatment of MDD deserves additional research.

The need to target exact cell types with a specific treatment, without changing the physiology of other cells or tissues, has been one of the major challenges during the most recent years. Furthermore, for *in vivo* application, there is also the need to develop transport systems that cross major biological barriers including the BBB. Under this concept, exosomes have been postulated as potential new delivery vehicles for specific miRNAs or their inhibitors (Kim et al., 2006; Bhatnagar et al., 2007; Lai et al., 2011; Yang et al., 2011; Hu et al., 2012; Ohno et al., 2013). Recent advances have been made to target the brain using a systemic delivery route and to specifically target immune cells in order to reduce neuroinflammation and treat associated disorders as MDD. A link between immune dysregulation and the pathophysiology of at least some forms of major affective disorder has long been hypothesized and abnormalities in peripheral cytokines in depressed patients have led to propose a primary immunological etiology for MDD (Eyre and Baune, 2012). While increased IL-6 was observed to dysregulate genes involved in miRNA machinery, IL-10 revealed neuroprotective properties in first episode psychotic patients with depression (Noto et al., 2015). A better understanding of the pathophysiology of MDD, mainly the immune mechanisms, may help in selecting more powerful biomarkers and discovering target effective treatments using exosomes as delivering vehicles.

Neurons use glial cell exosomes to improve stress tolerance by their role as multifunctional signal emitters. The low levels of serotonin found in MDD patients (Jacobsen et al., 2012) may compromise cell exosome release (Glebov et al., 2015), establishing a bridge between MDD and the potential role of exosomes in disease progression, either for not releasing adequate growth factors and neurotransmitters or by transporting inflammatory miRNAs. In addition, we should consider that exosomes represent a mechanism to get rid of toxic proteins having a neuroprotective action (Joshi et al., 2015), but can also be mediators of neuroinflammation (Gupta and Pulliam, 2014) by their cargo in miRNAs. Alvarez-Erviti et al. (2011b) provided the proof-of-concept that exosomes are a good delivery system of siRNAs to the mice brain. The Authors showed that intravenously administered exosomes derived from autologous murine dendritic cells are able to knockdown specific proteins in neurons, microglia, and oligodendrocytes in the brain of treated animals, proving the therapeutic potential of autologous exosome-based delivery system. Most attractive, Bryniarski et al. (2013) recently showed that exosomes can deliver their cargo into precise cell types in antigen-specific manner by expressing on their surface specific antibodies.

One preferential route of exosome administration into brain tissue is intranasal delivery. An initial study showed that exosomes derived from activated dendritic cells are efficiently delivered by intranasal administration. They showed to be preferentially taken up by oligodendrocytes and to improve baseline myelination (Pusic et al., 2014), thus suggesting to be a promising strategy for myelin-impaired disorders as MDD (Rajkowska et al., 2015). Interestingly, modified exosomes containing the signal transducer and activator of transcription 3 (Stat3) inhibitor JSI124, have previously been shown to be specifically delivered to microglia cells via the intranasal route, showing a significant protection from LPS-induced brain inflammation (Zhuang et al., 2011).

Most attractively, recent studies showed that intraperitoneal injection of the microglia inhibitor minocycline in adult mice partially prevents LPS (Henry et al., 2008) or Interferon-α-induced (Zheng et al., 2015) depressive-like symptoms. However, studies on the use of non-steroid anti-inflammatory drugs (NSAIDs; Jiang and Chang, 1999) or COX-1 inhibitor (Choi et al., 2009), being COX-1 mainly activated in microglial cells, reported an increase of depressive episodes in psychiatrically healthy individuals. Moreover, a single administration of LPS to severely depressed patients led to a short-term elevation of first-line cytokines improving the depressed state in the first 24 h (Bauer et al., 1995). This finding suggests that the loss of microglia proper function, which may be induced by a strong proinflammatory stimulus as LPS, may also be in the basis of depression-like symptoms. So, it may be hypothesized that deliver of microglia inhibitors or microglia inducers (depending on the immune activation state of the depressed patient) within vesicles targeting microglia may result in promising therapeutic strategies for MDD.

Overall, the production of EVs from patients’ own cells that may be engineered to load specific miRNAs cargos and express at cell surface known proteins to be recognized by specific cell types, may in the future be an ideal strategy for personalized autologous therapies. In addition, the induction of exosome release and cell-to-cell communication for transmission of cytoprotective signals may have additional benefits in some specific circumstances. One could imagine combined therapy content in EVs targeting many different sorts of natural MDD resistances at once. However, we must not forget that a great deal of work must be performed before such therapeutic approaches may become feasible in the clinic for neurodegenerative and psychiatric disorders, including MDD.

## Author Contributions

DB contributed to the conception, design, data collection and critical analysis of the literature, as well as to the writing of the review. AF have given a substantial contribution to data collection, critical analysis of the literature and writing of the review.

## Funding

Supported by the projects PTDC/SAU-FAR/118787/2010 (to DB), EXPL/NEU-NMC/1003/2013 (to AF) and in part, by iMed.ULisboa (UID/DTP/04138/2013), from Fundação para a Ciência e a Tecnologia (FCT). The funding organization had no role in study design, data collection and analysis, decision to publish, or preparation of the present manuscript.

## Conflict of Interest Statement

The authors declare that the research was conducted in the absence of any commercial or financial relationships that could be construed as a potential conflict of interest.
